# New Strategy for the Immobilization of Lipases on Glyoxyl–Agarose Supports: Production of Robust Biocatalysts for Natural Oil Transformation

**DOI:** 10.3390/ijms18102130

**Published:** 2017-10-12

**Authors:** César A. Godoy

**Affiliations:** Departamento de Química (LIBB), Grupo de Investigación en Ingeniería de los Procesos Agroalimentarios y Biotecnológicos (GIPAB), Universidad del Valle, C.P. 76001 Cali, Colombia; cesar.godoy@correounivalle.edu.co; Tel.: +57-2-3212148 (ext. 118)

**Keywords:** lipase, covalent immobilization, glyoxyl–agarose, nucleophilic catalysis, PUFAs (polyunsaturated fatty acids), biodiesel

## Abstract

Immobilization on Glyoxyl–agarose support (Gx) is one of the best strategies to stabilize enzymes. However, the strategy is difficult to apply at neutral pH when most enzymes are stable and, even when possible, produces labile derivatives. This work contributes to overcoming this hurdle through a strategy that combines solid-phase amination, presence of key additives, and derivative basification. To this end, aminated industrial lipases from *Candida artarctica* (CAL), *Thermomyces lunuginosus* (TLL), and the recombinant *Geobacillus thermocatenulatus* (BTL2) were immobilized on Gx for the first time at neutral pH using anthranilic acid (AA) or DTT as additives (immobilization yields >70%; recovered activities 37.5–76.7%). The spectroscopic evidence suggests nucleophilic catalysis and/or adsorption as the initial lipase immobilization events. Subsequent basification drastically increases the stability of BTL2–glyoxyl derivatives under harsh conditions (t_1/2_, from 2.1–54.5 h at 70 °C; from 10.2 h–140 h in 80% dioxane). The novel BTL2-derivatives were active and selective in fish oil hydrolysis (1.0–1.8 μmol of polyunsaturated fatty acids (PUFAs) min^−1^·g^−1^) whereas the selected TLL-derivative was as active and stable in biodiesel production (fatty ethyl esters, EE) as the commercial Novozyme^®^-435 after ten reaction cycles (~70% EE). Therefore, the potential of the proposed strategy in producing suitable biocatalysts for industrial processes was demonstrated.

## 1. Introduction

Microbial lipases have been extensively applied in several biotechnological processes including biofuel production and food biotechnology [1,2,3,4,5]. As with most enzymes, price is still a disadvantage for its industrial implementation when compared with conventional—but less green—catalysts [6,7]. Among other complementary strategies, lipase immobilization on mesoporous supports has been probed as an efficient tool to solve such limitations by allowing enzyme re-use and stabilization [6,8,9]. Applications of immobilized lipases include production of cocoa butter substitute; production of bioactive compounds, flavors, and fragrances; and enantioselective resolutions. The most used immobilized lipases are Lipozyme RM IM^®^, Lipozyme TL IM^®^, and Novozyme^®^ 435 [10,11,12,13]. The latter, based on CAL B (*Candida antarctica* lipase B) immobilized on the Lewatit^®^ VP OC 1600 support, is a paradigmatic example of how to obtain a biotechnological solution for the industrial production of biodiesel from vegetable oil [7,14,15].

Immobilization on Glyoxyl–agarose support (Gx) is recognized as one of the best strategies to stabilize enzymes, surpassing not only other immobilization approaches but also tools derived from molecular biology [16,17]. In some cases, optimal Gx enzyme derivatives can be up to 60,000-fold more stable than the free enzyme under very harsh conditions such as in high temperatures (>60 °C) or in concentrated inactivating organic solvents (>50% *v*/*v*) and, eventually, reactivation can be achieved [18,19]. Thus, immobilization on Gx provides an enormous application potential for white biotechnology especially under non-conventional reaction conditions [6,20]. 

However, immobilization on Gx cannot be performed at neutral pH when most enzymes are stable, constituting one of its crucial disadvantages [21]. Such a limitation relies essentially on: (i) the reversible nature of the imine bond formed between the enzyme and the support; (ii) the fact that equilibrium is shifted towards reactants (imine bond hydrolysis) since the immobilization media are essentially water; and (iii) the low reactivity at pH 7.0 of nucleophilic (primary amine) enzyme groups against the aldehyde groups of the Gx support [17,20]. Hence, the probability of immobilization on Gx increases in pH conditions where more enzyme nucleophilic groups are able to establish bonds with the support [17,22]. Since the most abundant enzyme nucleophile is epsilon-NH_2_ (pK_a_ 10.5) [16,17,20,23], immobilizations on Gx are usually performed at pH 10 or above. This implies that the applicability of Gx, with few exceptions (e.g., some multimeric proteins [24,25,26]), is limited to enzymes that must be not only rich in lysyl residues but also alkaline-tolerant [17,20,23]. 

Different approaches have been proposed to partially overcome such a limitation by increasing the reactivity of the enzymes with Gx at pHs lower than 10: (i) The chemical amination of enzymes with ethylenediamine in addition to increasing the number of available protein primary amine groups. This allows a decrease in the immobilization pH on Gx to 9.0, as the introduced chemical groups have a lower pKa than epsilon-NH_2_ (9.2 and 10.5, respectively) [27,28]. However, it cannot be applied to proteins which are unstable during the amination process or at pH ≥ 9.0, such as those applied during the immobilization of the resulting aminated enzymes [22,29]. (ii) The addition of reducing agents such as cyanoborohydride or aminoboranes in some cases decrease the immobilization pH on Gx below 9.0 by reducing the initial reversible enzyme–Gx imine bonds to irreversible secondary amino bonds. Nevertheless, as these toxic agents are not selective, they also reduce aldehyde glyoxyl groups which limits Gx reactivity during the immobilization process, producing poorly stabilized derivatives [30]. (iii) By contrast, the presence of thiolated agents (e.g., beta-mercaptoethanol, *N*-acetylcysteine, DTT, etc.) decreases immobilization pH on Gx to 8.0 for some enzymes (using DTT) without sacrificing support reactivity [31]. However, besides the relative toxicity and high cost of these agents (e.g., DTT), the strategy excludes proteins containing essential disulfide bridges that are prone to being broken by disulfide-exchange [32,33]. 

Hence, to extend the usefulness of Gx as an immobilization support for white biotechnology applications, it is necessary to find new strategies that surpass or complement those that currently exist. Those eventual new strategies may take advantage of key organic synthesis tools such as nucleophilic catalysis: diverse aniline and anthranilic acid (AA) derivatives have been used as organocatalysts for imine bond formation during the synthesis of oxime or hydrazone protein conjugates under soft conditions (neutral pH and room temperature) [34,35]. Surprisingly, its applicability in the context of covalent enzyme immobilization has not yet been assessed. 

Thus, the present work hypothesis is that such nucleophilic catalysts could also contribute to increase enzyme and Gx reactivity during imine bond formation. The idea was applied for the first time to native and aminated enzymes unable to immobilize at pH 7.0 on Gx, namely the recombinant lipase of *Geobacillus thermocatenulatus* (BTL2) and the industrial lipases of *Thermomyces lanuginosus* (TLL) and *Candida antarctica* lipase sp. 99–125 (CAL). The effects in the immobilization parameters and derivative stability of aminated and native enzymes of AA-related compounds and the reference additive DTT [31], and those of the initial and final immobilization pH, were studied. The spectroscopic properties (ATR-FTIR and UV-Vis) of selected lipase and Gx support derivatives were determined as an initial approximation to unveil the immobilization mechanism. The applicability of the biocatalysts obtained following the proposed strategy was also assessed in two reactions of industrial relevance: parameters representing the activity, selectivity, stability, and re-usability of selected derivatives were determined and compared against conventional Gx–BTL2 derivatives in sardine oil hydrolysis; for the production of biodiesel (EE) via transesterification of palm olein with ethanol using a novel Gx–TLL derivative, reaction parameters were compared with those of the conventional Gx–TLL derivative and the industrial reference biocatalyst Novozyme^®^435. 

## 2. Results and Discussion

### 2.1. Immobilization of Enzymes at Neutral pH on Gx: Suggested Mechanism of Gx Modification and Enzyme Immobilization with Additives

According to Table 1, in absence of additives or a previous lipase amination, the immobilization yields at pH 7 and 25 °C were below 40% even after 24 h; this is in concordance with previous results using DTT [31]. 

The selected immobilization additives present different effects on the immobilization yields (Table 1): MA was the less effective (28.0–44.3%) as determined in other imine-bond reactions [34], while DTT and AA allowed the highest immobilization yields (69.2–99.7%). DTT has been previously applied as an immobilization additive for other non-aminated enzymes [31] and functions as a reference in this work, while AA has the advantage of a relatively low toxicity and price among the related compounds assayed [37]. Therefore, these additives were used to assess other immobilization parameters.

As seen in Table 2, the activity of soluble BTL2 (residual control activity) remains above 90% regardless of the additive, whereas DTT was deleterious for TLL and CAL. The latter would rely on the contrasting effects of DTT on each enzyme: it preserves key Cys residues that would otherwise be involved in BTL2 deactivation [18] and can break structurally relevant disulfide bridges present in TLL or CAL [38,39]. This would also explain the fact that recovered activities (the activity expressed by the enzyme once immobilized [36]) were relatively low for CAL and TLL but high for BTL2 when using DTT as additive (Table 2). Hence, AA was selected as the optimal immobilization additive for TLL and CAL, while DTT was selected for BTL2.

**Figure 1:**
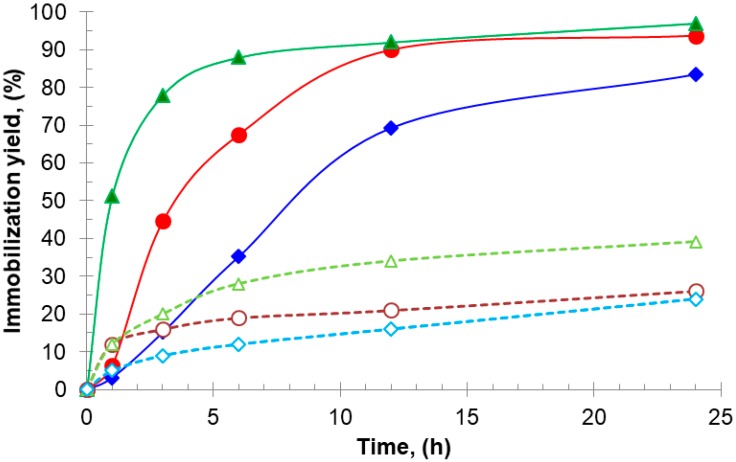
Immobilization progress of aminated lipases on glyoxyl–agarose support at 25 °C and pH 7.0: TLL without additives (▵) and with AA 20 mM (▴); BTL2 without additives (○) and (●) with AA 20 mM; CAL without additives (◊) and with DTT 20 mM (♦).

**Figure 2:**
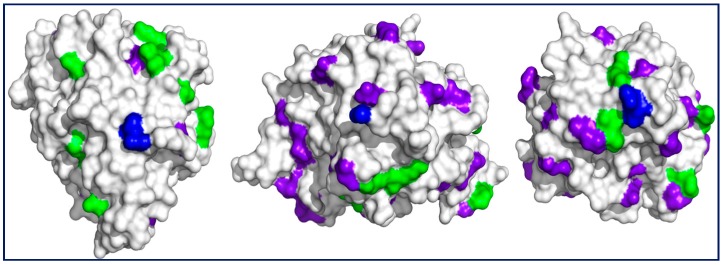
Surfaces of (left) CAL (PDB 5a71), (middle) BTL2 (PDB 2w22), and (right) TLL (PDB 1dte) centered at the (blue) N-terminus. Other surrounding residues which are potentially reactive at neutral pH with Gx are (green) lysyl ε-NH_2_ groups and those amino groups added upon chemical amination (purple-blue) of glutamyl and aspartyl residues. In all cases, the active center is on an enzyme surface projection different from the one shown here. Notice the lower number of reactive residues for CAL. Representations made with Pymol version 1.3.

On the other hand, the time taken to reach 50% of the 24 h immobilization yield (t_50%_) can be used as a measure of the immobilization rate: at pH 7.0, t_50%_ for aminated TLL, BTL2, and CAL using additives were higher (1 h, 3 h, and 7 h, respectively, Figure 1) than those obtained under conventional immobilization conditions (0.1 h, 0.2 h, and 0.5 h, respectively, data not shown), namely, at pH 9.0 without additives [27,28,29]. This reflects a decreased but sufficient availability of reactive amino groups against the Gx support at neutral pH (e.g., N-terminus and surrounding amino groups, Figure 2) when compared with that at pH 9.0, where groups such as those introduced after chemical amination with EDA are more reactive.

The lower rates (higher t_50%_) and yields of immobilization for CAL when compared with those of TLL and BTL2 would be a consequence of a lower number of CAL potentially reactive residues as evidenced in Figure 2.

**Figure 3:**
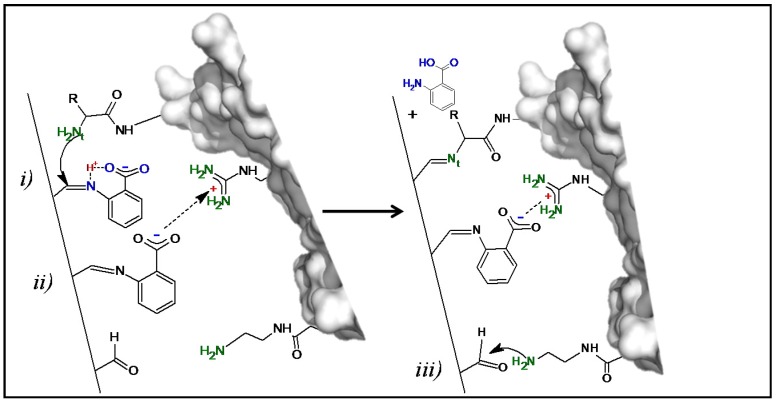
Proposed one-pot mechanism of the generation of a bifunctional Gx support and enzyme immobilization. (**Left**) Bifunctional surface formed after AA reaction with the Gx support; the immobilized additive molecules could interact with the enzyme through covalent nucleophilic catalysis [34,35] (i) and/or through cationic exchange (ii) triggering enzyme immobilization. (**Right**) The increased proximity between the immobilized enzyme reactive groups and the support promotes additional covalent bonds (iii) constituting a multipoint attachment immobilization. Representations made with Pymol version 1.3.

The proposed enzyme immobilization mechanism on Gx mediated by AA at pH 7.0 is depicted in Figure 3: initially, AA molecules react reversibly with the aldehyde groups of the support through its nucleophilic amino group establishing a bifunctional and dynamic support surface with carboxylate and remaining aldehyde groups. Then, by proton transfer through its *orto*-carboxylate group [34], the immobilized additive (upper AA molecule in Figure 3) acts as a catalyst by promoting the nucleophilic attack of the most reactive enzyme amino group—probably the N-terminus at neutral pH [31]—which results in the formation of an imine enzyme–support bond and the subsequent liberation of AA, ending the catalytic cycle; an akin mechanism for DTT as additive could also take place with one sulfhydryl group acting as an anchoring point and the other as a proton donor like the *orto*-carboxylate group of AA. 

However, the described catalytic effect would be one component of the whole action of the additive during the enzyme immobilization since, according to the spectroscopic evidence, the interaction of the support with the immobilization solution containing 20 mM AA at pH 7.0 implies modifications on the Gx surface that are not fully reversible as expected for a mere catalytic action: even after exhaustive washings of the resulting support with an immobilization solution lacking the additive (Section 3.2.1 and Section 3.7), both the UV-Vis and FTIR-ATR spectra evidence characteristic energy transitions differing from those of the plain Gx support and resembling those of AA.

**Figure 4:**
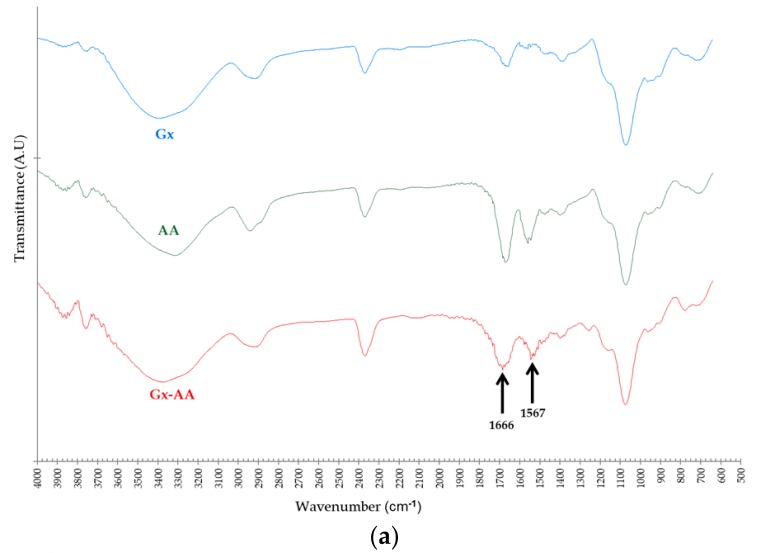

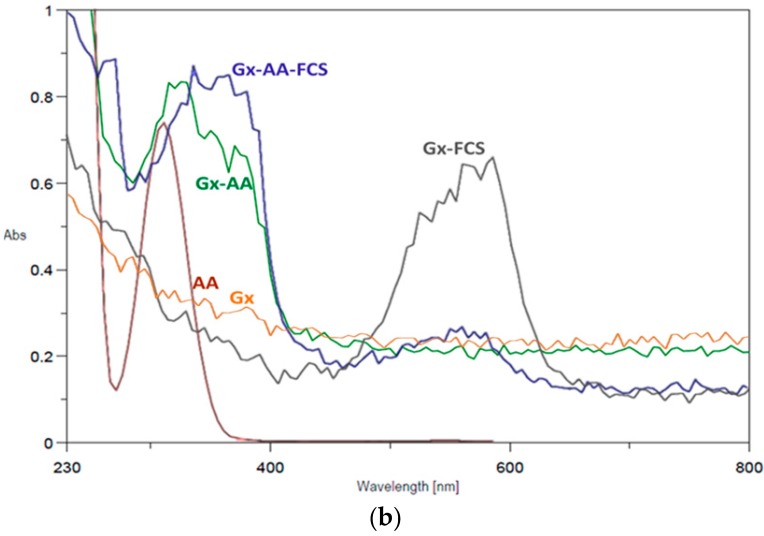
(**a**) Comparative FTIR-ATR transmittance spectra of Gx support derivatives: plain Gx support without modifications (Gx, blue), pure anthranilic acid (AA, green), and Gx anthranilic acid derivative support (Gx–AA, red); (**b**) Comparative UV-Vis spectra: free AA spectra (brown), plain Gx support (Gx, orange), Gx after treatment with 20 mM AA (green), Gx after fuchsine staining (grey), Gx after treatment with AA 20 mM and then stained with fuchsine (blue).

As seen in Figure 4a (FTIR-ATR spectra) the intensity ratio of the bands between 1650–1700 cm^−1^ (left arrow) to the bands between 3300–3400 cm^−1^ (O–H or N–H stretch bands) follow the order AA > Gx–AA > Gx; this could be the result of the presence of additional C=O bonds—and thus a higher probability of the respective asymmetric bond stretches [40]—from the carboxylic groups of the AA molecules that remain immobilized on the Gx support surface. However, a key distinguishing element for Gx–AA, in regard to the plain Gx support spectra, is the band between 1525–1575 cm^−1^ (right arrow) attributable to the C=C–N stretch of the aryl-imine keto form [41] of the additive linked to the support.

Regarding the UV-Vis spectra (Figure 4b), bands around 300–400 nm due to π→π* benzenoid transitions [42] are present for pure AA, Gx–AA and even for Gx–AA after treatment with fuchsine, the latter with an additional band between 450–650 nm in the visible region due to the π→π* transitions of the quinoid fuchsine ring [43]. As discussed for the respective FTIR-ATR spectra, this strongly suggests not only that AA is immobilized on Gx but also that there are aldehyde groups able to react with the dye as free groups (Figure 3) or after the displacement of immobilized AA molecules by fuchsine; the latter removes some, but not all, AA molecules since the UV-Vis spectra of the resulting support (Gx–AA–FCS, Figure 4b) still show an intense band between 300–400 nm. This suggests an intense interaction between AA and the support that is not easily broken by the reacting amino groups of the dye which mimic those of the protein in the experiment.

**Figure 5:**
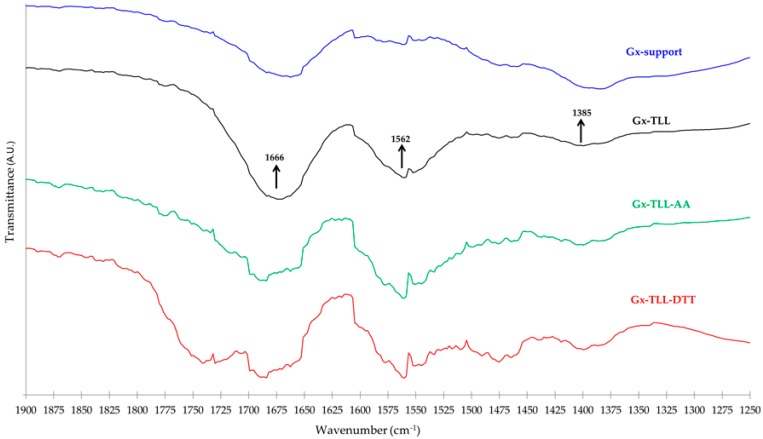
FTIR-ATR transmittance spectra within the distinctive region. Gx support without modifications (blue) and Gx biocatalysts resulting from the immobilization of TLL: at conventional pH 10 (black), in presence of 20 mM AA at pH 7.0 (green), or in presence of 20 mM DTT at pH 7.0 (red).

In Figure 5 is shown the IR region that presents the more marked differences between the Gx support and its different enzymatic derivatives. The latter present a band between 1500–1625 cm^−1^ that is absent in Gx. Since these bands coincide with the typical protein Amide II band (right arrow), it is very likely that they account for the immobilized enzyme. Even when it is not possible to clearly distinguish other remaining protein Amide bands because of overlapping with those attributable to H–O–H, O–H, C–O–C, and C=O bonds in the agarose matrix (bound water, alcohol and glycosidic groups, etc.) and the support surface (aldehyde) [44,45], it is very likely that the contribution of the Amide I band is responsible for the fact that in all TLL and Gx spectra derivatives, the relative size of the band near 1666 cm^−1^ is always higher than that of the band at 1385 cm^−1^.

According to the above spectroscopic evidence, since not all the additive molecules attached to the Gx surface are liberated from the support, it is expected that interactions other than catalytic between the enzyme and the generated bifunctional Gx support will be present. Indeed, as also seen in Figure 5, the FTIR-ATR spectra of the derivatives of Gx and TLL obtained in the presence or absence of DTT or AA show unique features especially where protein amide bands appear [5,46]. This suggests that the specific nature of the interactions between the enzyme and the immobilized additive (e.g., ionic for AA or disulfide exchange for DTT) influences the acquired protein structure after immobilization. Indeed, this may explain the different biocatalytic properties of the resulting lipase derivatives determined during natural oil transformations (Section 2.3 and Section 2.4).

Enzymes immobilized following the suggested mechanism (Figure 3) would produce highly stabilized derivatives given the multipunctual nature of the enzyme–support interactions; additionally, considering that after the additive action there would be unreacted aldehyde groups on both the protein surface and the support (as suggested by the UV-Vis results after fuchsine staining, Figure 4b), the number of covalent interactions could be further augmented by increasing the pH as in other Gx immobilizations [20]. The validity of these statements and the usefulness of the obtained derivatives were assessed in experiments, the results of which will be analyzed below.

### 2.2. Effect of the Initial and Final pH on Derivative Recovered Activity and Stability: Production of a Highly Stabilized Biocatalyst

Given their higher recovered hydrolytic activity and immobilization yield (Table 2) and the availability of previous reports of BTL2 immobilized on Gx under different conditions as references [22,27], the aminated BTL2 derivatives (immobilized at pH 7.0 or 8.0 with DTT as the optimal additive) were selected to study the effect of increasing the incubation pH as a post-immobilization treatment (basification) on derivative stability. Their stabilities were also compared against the classic reference BTL2-covalent CNBr and other Gx derivatives previously obtained through a similar procedure intended to increase enzyme–support bonding [22,31].

In general, high recovered activities were observed for the novel biocatalysts (Table 3): 72–76% and 62–65% for derivatives immobilized at pH 7.0 or pH 8.0, respectively, which are values close to that of the reference Gx derivative (64%). Thus, the initial immobilization pH impacts more markedly on the recovered activity than the following basification, as previously seen in other systems [22,31]. 

The comparatively higher recovered activities for derivatives resulting from immobilizations at pH 7.0 may reflect that the orientation of the enzyme on the support corresponds to surfaces far from the catalytic domain (Figure 2), namely, those comprising N-termini (Section 2.1). By contrast, immobilizations at pH 8.0 or above (as for the Gx reference derivative) may imply additional reactive amino groups (mainly protonated at neutral pH) such as those of chemically aminated Asp and Glu within the catalytic domain, increasing the probability of perturbing the catalytic activity, as suggested previously, during site-directed covalent immobilizations of the enzyme [22,36].

**Figure 6:**
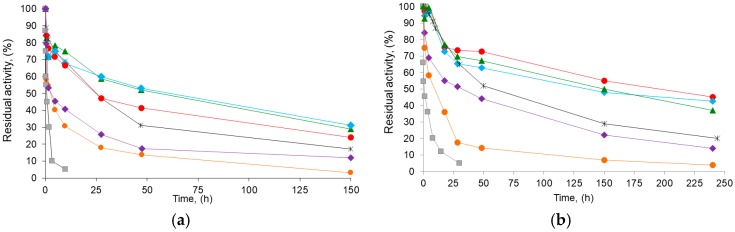
Time course inactivation of BTL2 biocatalysts: (**a**) inactivation at 75 **°**C, pH 7.0, and (**b**) in the presence of 80% (*v*/*v*) dioxane, 30 °C, and pH 7.0. CNBr-derivative of BTL2 (■) and glyoxyl derivatives of chemically aminated BTL2 immobilized with DTT: at pH 7.0 and incubated at pH 7.0 (●), pH 8.0 (▴), or pH 10.1. (♦); or immobilized with DTT at pH 8.0 and incubated at pH 8.0 (♦) or pH 10.1 (●). Reference Gx derivative immobilized at pH 9.0 and incubated at pH 10.1 in absence of DTT (*). The initial (100%) activity of enzyme derivatives was 5.0 IU/g. Error bars not shown for clarity (in all cases, S.D. below 5% units).

Table 3 also shows the stability parameters of the different BTL2 derivatives obtained. A typical course for a two-stage inactivation model (Figure 6; see also inactivation parameters in TS1) was observed as expected for immobilized enzymes [47,49]. In all cases, the different Gx derivative inactivation parameters at 70 °C or in dioxane 80% (*v*/*v*) indicated a higher stability than that of the reference aminated CNBr (e.g., up to 3.5-fold and 44-fold lower values for k_1_ and k_2_ in dioxane, respectively). Therefore, this suggests a higher number of enzyme–support bonds on the Gx derivatives (Figure 2) when compared with those on the CNBr derivative [22,27], which also agrees with the proposed multipunctual immobilization mechanism (Section 2.1).

On the other hand, regardless of the initial pH of immobilization or inactivation condition, increasing the post-immobilization pH (basification) always increased derivative stability (Table 3): e.g., the inactivation constants k_1_ and k_2_ of the BTL2 derivative immobilized at pH 7.0 and then incubated at pH 10.0 are two-fold and five-fold lower than those of the derivative immobilized and incubated at neutral pH. Thus, basification seems to promote a higher immobilized enzyme rigidity and thus a higher kinetic barrier for inactivation [22,27,29]. This suggests that around the vicinity of the initial anchoring point, there are enough aldehyde groups on the Gx derivative able to form additional bonds with the amino groups activated after basification (e.g., from lysyl and from other aminated acid residues inactive below that pH). This confirms that DTT does not compromise Gx reactivity, not only when BTL2 are immobilized at pH 8.0 [31], but also when the aminated enzyme is immobilized at neutral pH. 

It is important to note that the proposed strategy, namely, combining the solid-phase chemical amination, the presence of the additive during the immobilization at neutral or 8.0 pH, and a subsequent basification at pH 10, produces derivatives with an overall inactivation behavior that is better under the same inactivation conditions than that previously reported for BTL2 in most cases: the higher values of E_2_/E and, in some cases, lower values of k_2_ (inactivation in dioxane) make their t_1/2_ exceed those of the reference conventional Gx derivatives (up to 2.6-fold, Table 3) and around 6.5-fold higher than the t_1/2_ in dioxane for the most stable native BTL2 derivative obtained in the presence of DTT [31]. 

Therefore, it is very likely that these higher stabilizations are the result not only of aminated BTL2-support stabilizing interactions through enzyme surfaces prone to trigger inactivation by denaturants (e.g., areas comprising native Ser334 or Gln40 [22,36]), but also as the result of the positive effects of DTT on BTL2 activity [18]. 

### 2.3. Hydrolysis of Fish Oil

The obtained BTL2 derivatives were applied as the biocatalyst in the hydrolysis of fish oil (Table 4). The activity values (1.01–1.88 μmol min^−1^·g catalyst^−1^) were close to those of previously obtained BTL2 derivatives using other immobilization strategies [22]. As reported for other lipases in hydrolytic reactions, immobilization conditions influence derivative activity and selectivity [50,51]: the aminated enzyme immobilized at pH 8.0 with DTT and basified (at pH 10.1) results in the best biocatalyst in terms of selectivity, with values close to those of the aminated BTL2 mutant immobilized by site-directed multipoint covalent attachment at the surface comprising native Gln40 residue (eicosapentaenoic acid (EPA) to docosahexaenoic acid (DHA) ratios, EPA/DHA of 2.32 and 2.4, respectively) [22]. These properties, along with results concerning stability (Section 2.2), suggest that both derivatives may share a similar enzyme–support orientation and interaction strength, however, each derivative was obtained through different strategies which are much less simple than that presented here.

### 2.4. Ethyl Ester (EE) Production from Palm Olein

Low production of EE (<25%) was observed, even after 70 h, for all BTL2 and CAL covalent derivatives and for that of TLL immobilized in the presence of DTT whether dried or not (Table 5); for BTL2, this low transesterification activity was also seen when it was immobilized on poly-hydroxybutyrate beads [53], whereas the low EE yield seen for CAL and TLL derivatives produced by immobilizations mediated by DTT is probably related to the deleterious effect of the additive on enzyme activity, as discussed in Section 2. By contrast, the aminated TLL derivative obtained under classic immobilization conditions at pH 9.0/10.1 (51.6% EE_14 h_), the novel TLL derivative immobilized at pH 7.0 in presence of AA (71.5% EE_14 h_), and the commercial biocatalyst reference Novozyme^®^ 435 (75.1% EE_14 h_) were the most active biocatalysts after drying (Table 5). For the same derivatives but without the drying pretreatment, EE_14 h_ yield drops to 11.7%, 14.3%, and 47.2%, respectively, implying that a low water content is positive for the EE_14 h_ yield of all the biocatalysts, as determined for other systems where an excess of water may denote a competitive hydrolytic reaction [1,9]; it is also possible that the pretreatment with *tert*-butanol during drying (Section 3.5), as in other biocatalytic transesterifications [54], helps to improve the performance of TLL–Gx derivatives. It is very likely that the fact that Novozyme^®^ 435 results were less affected by moisture than TLL–Gx is due principally to the hydrophobic nature of its constituent support: Lewatit^®^ VP OC 1600 is based on a DVB-crosslinked polymer that probably excludes water and favors contact with the reacting oil in the microenvironment of the enzyme to a higher degree than the highly hydrophilic crosslinked- agarose matrix present in Gx–TLL derivatives.

Differences in terms of EE yield between Gx derivatives under the same enzyme and drying conditions may be due to the different protein structure acquired after immobilization (Section 2.1). For example, unlike the FTIR-ATR spectra of the more active Gx–TLL derivatives in EE production (reference and immobilized at pH 7.0 with AA, Table 5), the less active one (immobilized at pH 7.0 with DTT) has a band present between 1700–1800 cm^−1^ (Figure 5). This could be the result of a different local state of the peptide backbone [55,56] caused by DTT, which results in a less active enzyme structure in EE production after the immobilization process.

The fact that both the commercial catalyst and the TLL–Gx derivatives present conversions below 80% even after 44 h may be explained by taking into account chemical equilibrium considerations [1,9,15]: in the reactions conditions assayed herein, a stoichiometric and “greener” ethanol to oil molar ratio (3:1) was selected, while considerably higher ratios were necessary to obtain conversions above 90% using analog covalent TLL derivatives in other studies (9:1–18:1) [3,4]. 

Interestingly, the EE_14 h_ yield per mg of immobilized protein (BCA method, Section 3.4) for the more active covalent TLL derivative was 21.4, while for the commercial biocatalyst it was just 8.6. This higher specific activity for the novel biocatalyst implies savings in the quantity of enzyme needed which constitutes one of the main components of the cost for this kind of catalyst [9,15].

### 2.5. Operational Stability of Selected Biocatalysts

**Figure 7:**
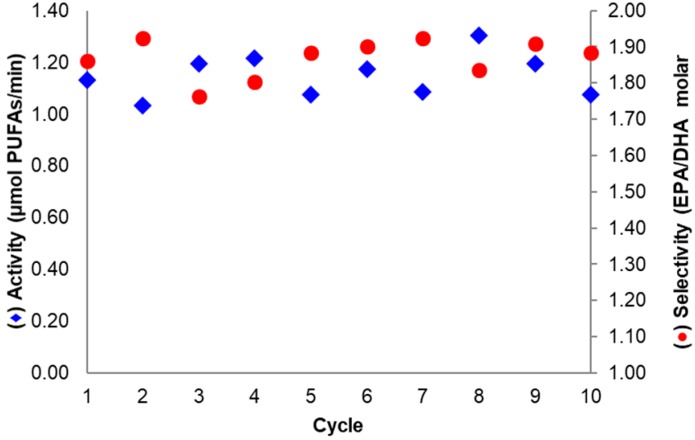
Sardine oil hydrolysis activity (◆) and selectivity (●) after successive reaction cycles. The Biocatalyst assayed was the most selective and was based on aminated BTL2 immobilized on Gx at pH 7.0 with 20 mM DTT and incubated at pH 10.1. Conditions according to Section 3.4 and Section 3.6.

The assessment of the operational stability of a biocatalyst is required to evaluate its implementation in industrial applications [6]. For the derivative where BTL2 was immobilized at pH 7.0 with 20 mM DTT and then basified (at pH 10.1), the activity and selectivity in sardine oil hydrolysis remained almost unchanged after ten reaction cycles (Figure 7). This was expected considering the high derivative stability observed under harsher inactivating conditions, as discussed in Section 2.2. 

**Figure 8:**
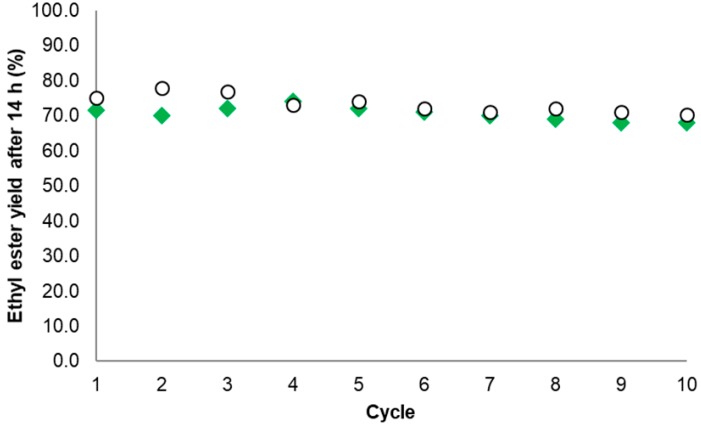
Ethyl ester yield (% *w*/*w*) after successive reaction cycles. Commercial reference Novozyme^®^ 435 (○) and the novel biocatalyst based on aminated TLL immobilized on Gx at pH 7.0 with 20 mM AA (◆**)**. Conditions according to Section 3.5 and Section 3.6.

In the case of EE production, the EE_14 h_ yield (%) for the best Gx derivative (obtained when TLL was immobilized in presence of 20 mM AA at pH 7.0) decreased from 71.5 to 68, thus retaining 95% of the initial activity. For the industrial reference Novozyme^®^ 435, the EE_14 h_ yield (%) decreased from 75.1% to 70.2%, retaining 93% of the initial activity (Figure 8).

Difficulties in the recovery of the biocatalyst particles were present especially for the agarose derivative given its smaller size when compared with Novozyme^®^ 435. Aside from the above, the novel TLL biocatalyst was as stable as the commercial reference under the same EE production conditions. Such a high stability of the EE_14 h_ yield for the novel Gx derivative is probably due to the intense interaction of the enzyme with the support within multipoint immobilizations [20,22,40] as expected for the proposed mechanism (Section 2.1). 

## 3. Materials and Methods

### 3.1. Materials

Cyanogen bromide activated Sepharose 4B (CNBr) and butyl Sepharose CL-8B were purchased from General Electric (Upsala, Sweden). Agarose 10BCL (50–150 μm) was purchased from Agarose Bead Technologies (Madrid, Spain). Fully activated glyoxyl–agarose 10BCL (150 μmol aldehyde groups/g) was prepared as previously described [30]. *Thermomyces lanuginosus* lipase (TLL), *Candida antarctica* lipase sp. 99–125 (CAL), 1,2-ethylenediamine (EDA), Ethanolamine hydrochloride, sodium metaperyodate, 1-ethyl-3-(dimethylaminopropyl) carbodiimide (EDC), Triton X-100, dithiotreitol (DTT), anthranilic acid (AA), methyl anthranilate (MA), aniline (AN), ethanol, *p*-nitrophenyl butyrate (*p*-NPB), docosahexaenoic acid (DHA), and eicosapentaenoic acid (EPA) and salts for buffering solutions were purchased from Sigma Chem. Co. (St. Louis, MO, USA). The sardine oil was a gift from BTSA, Biotecnologías Aplicadas, S.L. (Madrid, Spain); the Novozyme^®^ 435 was a gift from Novozymes (Bagsværd, Denmark). *Geobacillus thermocatenulathus* lipase 2 (BTL2) expressed in *E. coli* was produced, purified, and aminated in solid phase as previously described [22]. Other reagents and solvents were of analytical or HPLC grade. Novozyme^®^ 435 and Lewatit^®^ VP OC 1600 were kindly donated by Novozymes A/S and Lanxess^®^, respectively.

### 3.2. Production of Derivatives

#### 3.2.1. Immobilizations on Glyoxyl–Agarose Support (Gx)

Production of aminated lipases, CNBr, and Gx reference derivatives was performed as previously described [22,57,58]; soluble lipases were obtained by desorption from Lewatit^®^ VP OC 1600 (10 g) with 100 mL of 50 mM buffered solution, containing 5.0 mM EDTA, 0.5% (*w*/*v*) Triton X-100 (for CAL and BTL2), or 0.5% (*w*/*v*) CTAB (for TLL) for a duration of 1 h at 25 °C [27,28]. Aminated enzymes were then mixed with a suspension of 6.0 g of Gx support in 60 mL of 50 mM sodium phosphate buffer at pH 7.0 (and pH 8.0 for BTL2) with 20 mM of a given additive (fresh DTT, AA, MA, AN) and the mixture thermostated at 25 °C; when needed, the addition of NaBH_4_ at 5 mg/mL for a 30 min duration defined the end-point of the enzyme immobilization process [31]; the resulting Gx derivatives were washed ten times with 50 mL of 25 mM sodium phosphate buffer at pH 7.0 and stored at 4 °C until further use.

To avoid mass-transfer artifacts, derivatives were obtained by adding around 5.0 IU *p*-NPB hydrolytic activity/g for stability determination [27,36]; for applications in palm olein transesterification and sardine oil hydrolysis, highly loaded derivatives were obtained by adding 100 IU *p*-NPB hydrolytic activity/g of support [22]. 

#### 3.2.2. Basification: Incubation at Higher pH of DTT-Immobilized BTL2 on Gx

BTL2 derivatives (0.4 g) obtained after 12 h of interaction with Gx and DTT (at pH 7.0 or 8.0) were filtered, washed ten times with 4 mL of the respective immobilization buffer, and mixed for an additional 12 h with 4 mL of a buffer containing 5 mM EDTA with the same or higher pH of immobilization (*basification*) to promote additional enzyme–support bonding [22,27]: pH 7.0 or 8.0 with 50 mM sodium phosphate buffer or pH 10.1 with 50 mM sodium bicarbonate buffer. Then, NaBH_4_ was added as described in Section 3.2.1 [31]. Finally, the derivatives were washed thoughtfully with 50 mM sodium phosphate at pH 7.0 and stored at 4 °C until further use [17,20,22].

### 3.3. Derivative Stability

The derivative *residual activity* is expressed as the remaining percentage of the t_0_ activity at a given incubation time under deactivating conditions [22]. Half-life time (t_1/2_) was determined by using a nonlinear fit of the residual activity (%) versus time [47,49]. *Stabilization factors* were calculated as the ratio of t_1/2_ of a given BTL2 derivative to the t_1/2_ of the respective control CNBr derivative [22,48]. The inactivation course of the derivatives at 70 °C, 50 mM sodium phosphate and pH 7.0, or in dioxane/25 mM Tris-HCl aqueous solution (80:20 *v*/*v*) pH 7.0 and 25 °C, was conducted as previously described [22,27].

### 3.4. Hydrolytic Activity and Protein Determination

Esterase activity of soluble or immobilized enzymes against *p*-NPB and the lipolytic activity and selectivity (EPA/DHA molar ratio) against sardine oil for BTL2 were assayed at pH 7.0 and 25 °C as described before [52]. Proper controls and dilutions of DTT (<5 mM) containing solutions must be done in order to avoid interference in the *p*-NPB assay; one international unit (IU) is defined as the amount of enzyme required to hydrolyze 1 μmol of *p*-NPB min^−1^ [27].

Protein determination was performed according to the Protein Assay Kit protocol at 60 °C and for 30 min using BSA as a standard (Pierce^®^ BCA). When DTT was present, Compat-AbleTM protocol was used. The amount of protein was measured in proper dilutions of filtered aliquots of control and immobilization supernatants after the decantation of the support. The protein loading on the supports was calculated from the difference in protein content measured between the control and the respective immobilization supernatant after 24 h.

### 3.5. One-Step Solvent-Free Ethyl Ester Production from Palm Olein

Around 70 mg (wet basis) of selected lipase derivatives or Novozyme^®^ 435 (as commercial reference) were used as produced or after this *drying procedure*: derivatives were mixed with 200 μL of *tert*-butanol for five minutes and the supernatant was discarded; this was repeated thrice [54]. Afterwards, derivatives were heated for 1.5 h at 40 °C or until achieving constant mass.

A mass of 70 mg of derivatives (wet basis, equivalent to 6% of the oil mass) was added (whether dried or not) to 1.17 g of palm olein and ~190 mg of absolute ethanol (ethanol:oil molar ratio 3:1) in hermetic vials; the vials were placed on a Thermomixer^®^ at 46 °C and 1700 rpm. Samples of 50 μL were withdrawn at different times and the content of ethyl esters (EE) analyzed using a FTIR-ATR methodology [59].

### 3.6. Operational Stability of Selected Derivatives

After each sardine oil hydrolysis reaction cycle (18 h), derivatives were collected by filtration, washed thoughtfully with distilled water, and re-used following the procedure previously reported [51,52].

For EE production, derivatives were collected after each reaction cycle (14 h) from the reaction medium by filtration, washed with *tert*-butanol, dried, weighed, and re-used following the procedure described in Section 3.5 [54]. The masses of all the reaction components were increased four times in order to make the recovery of the biocatalysts easier.

### 3.7. Spectroscopic Measures of Modified Gx Supports

For UV-Vis measures, Gx derivatives (0.2 g) were previously washed and suspended in 2 mL of deionized water; when needed, 50 μL of Schiff’s reagent was added and the suspension mixed for a duration of 30 min. Then, the solids were washed ten times, initially with 2 mL of a solution containing sodium bisulfite (0.58%) and acetic acid (3%), and then with deionized water. The stained solids were filtered, resuspended (0.15 g in 1 mL of deionized water), and the absorption spectra of the magnetically agitated suspension collected at 25 °C from 190–1100 nm in a Genesys Biomate 3S^®^ spectrophotometer with Peltier accessory.

For FTIR-ATR measures, different Gx derivatives (0.2 g) were washed ten times with 10 mL immobilization solution lacking additives and enzymes and then with deionized water, then filtered and dried at 30 °C under vacuum overnight until obtaining constant mass. FTIR-ATR spectra of the dried Gx derivatives and for pure DTT and AA were recorded using a Perkin Elmer Spectrum (with the Spectrum^TM^ Software) from 600–4000 cm^−1^ with 25 scans and 4 cm^−1^ resolution and an ATR probe with a cleaned diamond 3-reflection plate at the highest pressure for the Clamp (Pike Miracle^TM^ technologies). Normalization and ATR correction were performed using the Spectrum Software [46]. 

### 3.8. Statistical Analysis

All experiments were performed in triplicate. Significant differences among means were evaluated by using an ANOVA procedure (*p* < 0.05).

## 4. Conclusions

The aim of producing useful Gx derivatives at neutral pH was achieved by the strategy proposed in this study: solid-phase chemical modification of enzymes, the selection of adequate immobilization additives, and a later basification, contribute to obtaining active and robust biocatalysts in natural oil transformation. Thus, BTL2 derivatives were not only more stable but also as active and selective as the conventional Gx–agarose counterparts in sardine oil hydrolysis; TLL immobilized at pH 7.0 with AA on Gx produces a biocatalyst not only more active than its conventional analog but also with a performance comparable with that of the commercial reference Novozyme^®^ 435 in palm olein transesterification.

Recently, a strategy was proposed to obtain covalent enzyme derivatives using heterofunctional glutaraldehyde supports at neutral pH without the need for enzyme amination [60]. However, even when that point may be improved by optimization of the strategy presented in this paper, as it is, it allows the production of Gx derivatives with stabilization factors at 70 °C up to 15-fold higher and, yet, without the need for additional experimental steps to introduce new groups on the support. 

Hence, this work establishes a basis for further research aiming to add evidence to the proposed immobilization mechanism, as the fundament of the here-described optimizable one-pot alternative able to produce new heterofunctional Gx supports while promoting enzyme immobilization through nucleophilic catalysis and/or adsorption.

## Figures and Tables

**Table 1:** Immobilization yield of aminated lipases in presence of different additives at neutral pH and 25 °C.

Enzyme	Additive (20 mM)	Yield at 24 h (%) ^a^
TLL	-	39.1 ± 2.2
	DTT	99.7 ± 3.6
	AA	96.8 ± 2.7
	MA	44.3 ± 1.1
	AN	95.6 ± 3.0
CAL	-	23.9 ± 1.1
	DTT	88.5 ± 2.5
	AA	83.5 ± 2.3
	MA	32.0 ± 1.0
	AN	90.3 ± 3.1
BTL2	-	26.1 ± 0.9
	DTT	93.0 ± 4.1
	AA	69.2 ± 2.4
	MA	28.0 ± 0.4
	AN	72.0 ± 2.3

^a^ Immobilization yield in presence of DTT (dithiothreitol), AA (anthranilic acid), MA (methyl anthranilate), and AN (aniline) was calculated as 100% (1 − P_SI_ × P_CS_^−1^), where P_SI_ and P_CS_ indicate the total protein content after 24 h in the supernatants that were in contact before the measures with Gx–agarose and with non-activated agarose, respectively [36]. Total protein 2.12, 1.28, and 2.47 for BLT2 (*Geobacillus thermocatenulatus* lipase 2), TLL (*Thermomyces lunuginosus* lipase) and CAL (*Candida antarctica* lipase sp. 99–125), respectively, equivalent to 30 IU of p-NPB (*p*-Nitrophenyl butyrate) added to 6 g of support.

**Table 2:** Immobilization parameters of aminated lipases on Gx at neutral pH and 25 °C.

Gx-Derivative		Immobilization Parameters	
Enzyme	Additive (20 mM)	Residual Control Activity (%) ^a^	Recovered Activity (%) ^b^
TLL	-	96.3 ± 3.1	-
	DTT	5.1 ± 0.3	10.5 ± 0.4
	AA	55.2 ± 1.5	58.9 ± 1.7
CAL	-	94.7 ± 2.7	-
	DTT	64.4 ± 1.8	39.7 ± 1.1
	AA	83.2 ± 2.3	50.2 ± 2.0
BTL2	-	95.0 ± 2.0	-
	DTT	91.1 ± 1.6	76.7 ± 3.3
	AA	95.4 ± 3.0	72.1 ± 2.8

^a^ Residual control activity is defined as the percentage difference between the activity of the immobilization control (enzyme solution mixed with non-activated agarose) after 24 h and that of the control at the initial immobilization time (5 IU/g support) at 25 °C and pH 7.0 [27]. ^b^ Recovered activity is defined as the percentage ratio of the specific derivative hydrolytic *p*-NPB activity (IU/mg of immobilized protein) to that of the soluble enzyme (IU/mg free enzyme) under standard conditions [36]. Values are the mean of three different experiments where the standard deviation was never >5% of the mean value.

**Table 3:** BTL2 immobilization on Gx in presence of 20 mM DTT (except for the references) and derivative properties.

Derivative Production Conditions		Immobilization Parameters		Stability Parameters ^c^		Stabilization Factor ^d^	
Immobilization Step (pH)	Incubation Step (pH)	Yield (%) ^a^	Recovered Activity (%) ^b^	Half-Life at 70 °C (h)	Half-Life in 80% Dioxane (h)	At 70 °C	In 80% Dioxane
7.0	7.0	90	76	2.1	10.2	42	167
7.0	8.0	90	74	50.0	130	1000	2131
7.0	10.1	90	72	54.5	140	1090	2295
8.0	8.0	96	65	3.7	8.1	74	133
8.0	10.1	99	62	53.7	195	1074	3197
Reference Gx derivative (pH 9.0)	10.1	96	64	24.1	74.2	482	1216
Reference CNBr derivative (pH 7.0)	-	99	78	0.050	0.061	1	1

^a^ Immobilization yield was calculated as 100% (1 − P_SI_ × P_CS_^−1^), where P_SI_ and P_CS_ indicate the total protein content after 24 h in the supernatants that were in contact at neutral pH and 25 °C before the measures with Gx–agarose and with non-activated agarose, respectively. ^b^ Recovered activity is defined as the percentage ratio of the specific derivative hydrolytic *p*-NPB activity (IU/mg of immobilized protein) to that of the soluble enzyme (IU/mg free enzyme) under standard conditions [36].^c^ Half-life times at pH 7.0 at 70 °C or in 80% dioxane at 30 °C, according to the two-phase deactivation model [47]. ^d^ Stabilization factors were calculated as the ratio of t_1/2_ of a given BTL2 derivative and the t_1/2_ of the respective control CNBr derivative [22,48].

**Table 4:** BTL2 immobilization on Gx and derivative properties.

Derivative Production Conditions		Hydrolysis of Sardine Oil	
Immobilization Step (pH)	Incubation Step (pH)	Activity ^a^	EPA/DHA Ratio ^b^
7.0	7.0	1.38 ± 0.12	1.90 ± 0.10
7.0	8.0	1.22 ± 0.05	1.83 ± 0.05
7.0	10.1	1.13 ± 0.08	1.86 ± 0.03
8.0	8.0	1.01 ± 0.08	2.11 ± 0.02
8.0	10.1	1.04 ± 0.04	2.32 ± 0.15
Reference Gx derivative (pH 9.0) ^c^	10.1	1.10 ± 0.07	1.70 ± 0.07
Reference CNBr derivative (pH 7.0) ^d^	-	1.88 ± 0.09	1.97 ± 0.10

^a^ Activity is defined as the μmol of PUFAs (polyunsaturated fatty acids) = EPA (eicosapentaenoic acid) + DHA (docosahexaenoic acid), produced per minute and gram of derivative with 1 mg of protein in the biphasic system: 5 mL of cyclohexane, 5 mL of buffer tris–HCl 0.1 M at pH 7.0, and 0.50 mL of sardine oil at 25 °C [52]. ^b^ Selectivity is defined as the molar ratio of EPA to DHA produced after 24 h of reaction [52]. ^c^ Derivative obtained after the initial immobilization of aminated BTL2 in absence of DTT for a duration of 1 h at pH 9.0 followed by an incubation step at pH 10.1 for a duration of 24 h. ^d^ Derivative obtained after chemical amination of the CNBr derivative of native BTL2 immobilized at pH 7.0 and 4.0 °C. Values are the mean of three different experiments where the standard deviation was never >5% of the mean value (here not shown for clarity).

**Table 5:** Ethyl ester production catalyzed by novel and reference biocatalysts.

Derivative		Ethyl Ester (EE)			
Enzyme	Immobilization Condition	Yield at 14 h (%) ^a^		Yield at 44 h (%) ^a^	
		*Non-Dried*	*Dried*	*Non-Dried*	*Dried*
**TLL**	Reference	11.7 ± 1.2	51.6 ± 2.2	30.3 ± 1.1	69.8 ± 1.8
	DTT	5.3 ± 0.3	6.3 ± 0.5	5.7 ± 0.3	7.4 ± 0.3
	AA	14.3 ± 0.8	71.5 ± 2.8	23.2 ± 1.8	74.8 ± 2.8
**CAL**	Reference	8.0 ± 0.1	13.0 ± 0.9	8.5 ± 0.4	22.0 ± 1.7
	DTT	5.9 ± 0.5	5.3 ± 0.7	6.1 ± 0.3	7.2 ± 0.5
	AA	6.4 ± 0.4	5.6 ± 0.3	8.5 ± 0.6	7.7 ± 0.2
**BTL2**	Reference	0.9 ± 0.2	8.8 ± 0.2	2.0 ± 0.5	16.1 ± 0.1
	DTT	2.1 ± 0.1	9.5 ± 0.7	3.1 ± 0.8	15.0 ± 0.8
	AA	1.9 ± 0.2	10.3 ± 0.6	2.8 ± 0.1	11.2 ± 0.5
**Novozyme^®^ 435** (industrial reference [7,14,15])		47.2 ± 0.2	75.1 ± 2.9	78.0 ± 3.5	79.4 ± 1.9

^a^ Yield is defined as weight percentage (%) of ethyl esters (EE) in the oil phase after 14 h or 44 h of a reaction mixture that initially had 3:1 mol EtOH: mol palm olein and 5% (p/p) of biocatalyst at 46 °C and 1700 rpm.

## Supplementary Materials

Supplementary Materials can be found at http://www.mdpi.com/1422-0067/18/10/2130.
