# Supplementary material for: New Strategy for the Immobilization of Lipases on Glyoxyl–Agarose Supports: Production of Robust Biocatalysts for Natural Oil Transformation
Source: Int J Mol Sci. 2017 Oct 12;18(10):2130. doi: 10.3390/ijms18102130 (PMC5666812; doi:10.3390/ijms18102130)
Supplement: Supplementary file 1 [file ijms-18-02130-s001.pdf]

Supplementary material.

Table S1 inactivation constants for BTL2 derivatives immobilized in presence of 20 mM DTT except for the References.

Derivative Production Conditions		At 75 °C[a]				In dioxane 80%[a]			
Immobilization step (pH)	Incubation step (pH)	**k_1** (h^{-1})	**k_2** (h^{-1})	**E_1/E**	**E_2/E**	**k_1** (h^{-1})	**k_2** (h^{-1})	**E_1/E**	**E_2/E**
7.0	7.0	1.18 x 10	3.77×10^{-2}	7.52×10^{-1}	1.52×10^{-1}	3.64	6.12×10^{-2}	5.44×10^{-1}	6.87×10^{-2}
7.0	8.0	1.18 x 10	1.94×10^{-2}	4.57×10^{-1}	4.14×10^{-1}	7.51	1.74×10^{-2}	8.28×10^{-1}	2.49×10^{-1}
7.0	10.1	1.18 x 10	3.04×10^{-2}	9.87×10^{-1}	4.57×10^{-1}	1.87	1.28×10^{-2}	7.57×10^{-1}	2.34×10^{-1}
8.0	8.0	1.18 x 10	2.39×10^{-2}	7.96×10^{-1}	2.16×10^{-1}	1.15	3.72×10^{-2}	4.86×10^{-1}	1.15×10^{-1}
8.0	10.1	1.18 x 10	2.19×10^{-2}	9.92×10^{-1}	4.92×10^{-1}	2.10	2.67×10^{-2}	7.74×10^{-1}	2.34×10^{-1}
Reference Gx derivative (pH 9)	10.1	1.18 x 10	1.96×10^{-2}	1.02	2.17×10^{-1}	1.91	3.01×10^{-2}	8.41×10^{-1}	1.58×10^{-1}
Reference CNBr derivative (pH 7.0)	-	2.20 x 10	1.44×10^{-1}	5.60×10^{-1}	4.89×10^{-2}	4.03	5.70×10^{-1}	6.07×10^{-1}	4.89×10^{-2}

[a] Values according to the best-fit model (two-stage inactivation with residual activity); k_1 and k_2 are kinetic constants for the first-order inactivation rate constants for the first and second step respectively; E_1/E and E_2/E are the fractions of the initial enzyme specific activity (E) for the forms E_1 and E_2 resulting from each inactivation step [47,49].
